# U-Net-Based Segmentation of Microscopic Images of Colorants and Simplification of Labeling in the Learning Process

**DOI:** 10.3390/jimaging8070177

**Published:** 2022-06-23

**Authors:** Ikumi Hirose, Mari Tsunomura, Masami Shishikura, Toru Ishii, Yuichiro Yoshimura, Keiko Ogawa-Ochiai, Norimichi Tsumura

**Affiliations:** 1 Division of Creative Engineering, Graduate School of Science and Engineering, Chiba University, Chiba 263-8522, Chiba, Japan; mari-2d2@chiba-u.jp; 2 Central Research Laboratories, DIC Corporation, 631, Sakado, Sakura 285-8668, Chiba, Japan; masami-shishikura@ma.dic.co.jp (M.S.); toru-ishii@ma.dic.co.jp (T.I.); 3 School of Engineering, Chukyo University, Nagoya 466-0825, Aichi, Japan; yysmr13@gmail.com; 4 Kampo Clinical Center, Department of General Medicine, Hiroshima University Hospital 1-2-3, Kasumi, Minami-ku, Hiroshima 734-8551, Hiroshima, Japan; ikkandoo@gmail.com; 5 Graduate School of Engineering, Chiba University, Chiba 263-8522, Chiba, Japan; tsumura@faculty.chiba-u.jp

**Keywords:** U-Net, deep learning, segmentation, labeling, colorants, particle size distribution

## Abstract

Colored product textures correspond to particle size distributions. The microscopic images of colorants must be divided into regions to determine the particle size distribution. The conventional method used for this process involves manually dividing images into areas, which may be inefficient. In this paper, we have overcome this issue by developing two different modified architectures of U-Net convolution neural networks to automatically determine the particle sizes. To develop these modified architectures, a significant amount of ground truth data must be prepared to train the U-Net, which is difficult for big data as the labeling is performed manually. Therefore, we also aim to reduce this process by using incomplete labeling data. The first objective of this study is to determine the accuracy of our modified U-Net architectures for this type of image. The second objective is to reduce the difficulty of preparing the ground truth data by testing the accuracy of training on incomplete labeling data. The results indicate that efficient segmentation can be realized using our modified U-Net architectures, and the generation of ground truth data can be simplified. This paper presents a preliminary study to improve the efficiency of determining particle size distributions with incomplete labeling data.

## 1. Introduction

The products around us have different material appearances. A key element pertaining to the appearance of materials is the colorant. Colorants represent mixtures of pigments and resins, which are observed as particles in the form of lumps. Extensive research has been conducted on determining the correlation between color and particle size in the fields of food [1], medicine [2], and so on. In the measurement of seed characteristics, which is important for grain research, the analysis of the grain size and color information presents a deeper understanding of the raw material [1]. Kilcast et al. analyzed the effect of particle size and concentration on the perceived creaminess containing solid particles [3]. The study concluded that the creaminess decreased with the increase in particle size. The particle size distribution [4,5] influences the texture. As an example, when the particles become larger, the texture becomes rougher and grayer. Conversely, when there are fewer large particles and more small particles, the texture becomes finer and more jet-black. Therefore, it is necessary to determine the particle size distribution to implement the desired texture. Among various methods for determining the particle size distribution, a representative method involves the use of microscopic images. In this approach, the particle area in the microscopic images of colorants must be segmented [6,7,8].

Conventional segmentation methods involve manual labeling of the particle regions, which is time-consuming and inefficient. To address this problem, this study aimed to establish a highly accurate segmentation method based on multiple U-Net-type deep convolutional networks. Among the particles in the color microscope image, some particles have extremely dark interiors. The region-segmentation accuracy of these particles is lower than that of other particles. In this study, we also considered improving the accuracy of region segmentation for such particles. In general, the learning process of a U-Net-type deep convolutional network necessitates the preparation of datasets. However, the process of generating the ground truth labeled data in the dataset is time-consuming.

To address this problem, in this study, learning was implemented using incomplete labeling data. Moreover, we attempted to simplify the generation of ground truth data.

## 2. Materials and Methods

### 2.1. Datasets

**Figure 1:**
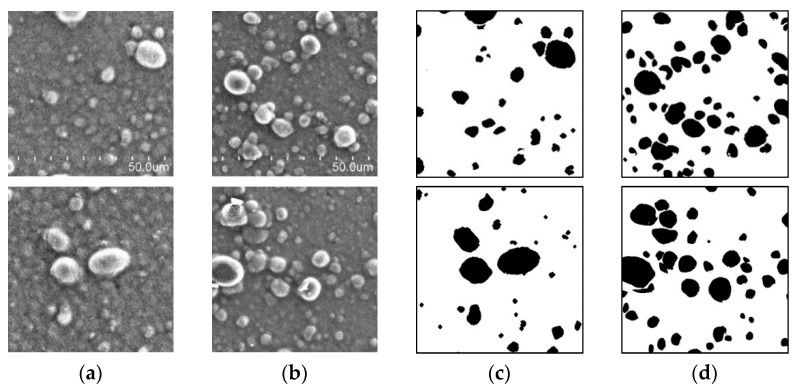
Sample dataset. Input data for a (**a**) small and (**b**) large number of particles. Output data for a (**c**) small and (**d**) large number of particles.

Training datasets include training data and ground truth data, and testing datasets include test data and ground truth data. The training and test data, shown in Figure 1a,b, respectively, corresponding to art papers printed using ink mixed with carbon black pigment and resin by a proofer, were extracted using a scanning electron microscope. The test data and correct-answers data shown in Figure 1c,d were generated by manually labeling the image shown in Figure 1a,b by using the CLIP STUDIO image editing software. The classification of “a small number of particles” and “a large number of particles” in Figure 1a–d is due to differences in the composition of the pigments and resins in each color material. In this study, our partners in the DIC corporation empirically divided the datasets into two classes based on the number of particles: small and large. Both images with a small number of particles and those with a large number of particles use the same carbon black colorant, but the difference is caused by the different pigment to resin ratios. As for whether the particles are small or large in number, in this study, we made an empirical judgment based on appearance. We used 60 images for training and 30 images for testing and evaluation. We employed ImageDataGenerator, a library in Keras, which performs data augmentation through artificial processing and synthesis. The dataset was expanded by sliding up and down, left and right, rotating the image, vertically flipping the image, changing the tilt, and randomly expanding the image horizontally and vertically based on the values of the empirically set parameters. The training data used to train the model included 2592 images after being augmented from 60 images, and test data comprised 30 images. The dataset was controlled to keep the differences within a certain range because the images were acquired by a procedure based on the work rules normally practiced at the DIC Corporation. This imaging procedure is not disclosed to the public because it is proprietary information; however, the quality is constant because it is routine work. The number of epochs is 10, and the batch size is 2.

### 2.2. Architecture of U-Net-Based Deep Convolutional Networks

The U-Net architecture is commonly used for segmenting different types of images. Therefore, it was considered a promising candidate for segmenting the microscopic images of colorants in this study [9,10,11].

**Figure 2:**
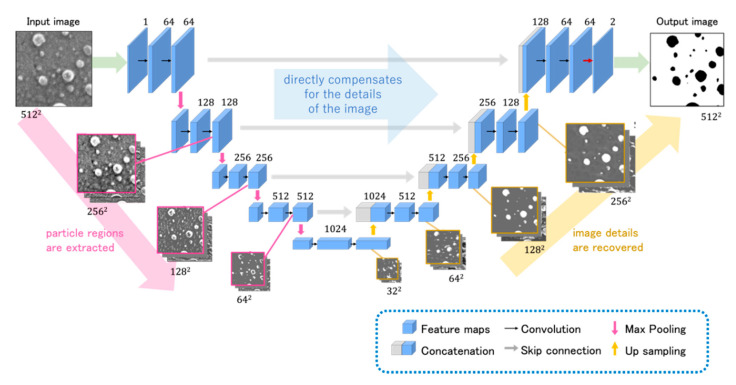
Conventional U-Net.

Section 2.2 presents the basic network architecture of U-Net [12]. The basic network architecture of U-Net is shown in Figure 2. The network structure based on U-Net consists of an encoder path and decoder path. The feature map, which is the result of learning at each layer in the encoder path, has skip connections that transfer the feature map to the layer of the same depth in the decoder path. This allows up-sampling to be performed without losing detailed information. Each pass contains a block of convolutional layers for learning. In the network structure used in this study, convolutional layers with a filter size of 3 × 3 were used. The encoder pass uses a max pooling layer with a filter size of 2 × 2 to reduce the image size from 512 × 512 to 32 × 32. These processes allow features to be extracted from the image. In this study, the features are the particles in the microscopic images of colored materials because the objects of region segmentation are the particles in the images. On the encoder side, particle regions are extracted by repeating the convolution and max pooling processes. In the decoder pass, the resolution is increased from 32 × 32 to 512 × 512 using an up-sampling layer with a filter size of 2 × 2. These processes are used to recover the image details. In doing so, the skip connections, which are a U-Net feature, directly compensate for the detailed image information lost when the image is scaled down at the encoder side. This process enables precise domain segmentation. Zero padding is used to match the image size on the encoder and decoder sides. Because zero padding is used, the output dimensions of all convolution layers for both down-sampling and up-sampling paths are identical, allowing information to be directly combined with skip connections. The number of channels increases from 1 to 1024 for down-sampling and decreases from 1024 to 2 for up-sampling.

We used Python to implement the U-Net algorithms for the training. The libraries used for the implementation in our model training are version 2.2.4 of Keras and version 1.11.0 of TensorFlow. Additionally, MATLAB was used to create the training dataset with incomplete labeling data, which is discussed in Section 2.4.

We used the rectified linear unit (ReLU) in every block of the convolutional layers and only used the sigmoid function in the output layer. ReLU is characterized by its low susceptibility to gradient loss problems and high processing speed as it is a simple function. We used the Adam optimizer for optimization. First, the gradient $g_{t}$ at time t was calculated as in the following equation, where $f\left(\theta \right)$ is the stochastic objective function with parameter θ, sets the weight to be optimized.

(1) $$g_{t}=\nabla_{\theta }f_{t}\left(\theta_{t}-1\right)$$

Exponential smooth moving average (EMA) is then used to find the first and second moments $m_{1}$ and $m_{2}$ of the gradient using the following equations, where $\beta_{1}$ and $\beta_{2}$ are the hyper parameters that control the decay rate of the moving average.

(2) $$m_{t}=\beta_{1}m_{t-1}+\left(1-\beta_{1}\right)g_{t}$$

(3) $$v_{t}=\beta_{2}v_{t-1}+\left(1-\beta_{2}\right)g_{t}{}^{2}$$

Using the values obtained in the above equation, we estimate the bias-corrected first-order moment $\hat{m_{t}}$ and second-order moment $\hat{v_{t}}$ as follows:

(4) $$\hat{m_{t}}=\frac{m_{t}}{1-\beta_{1}{}^{t}}$$

(5) $$\hat{v_{t}}=\frac{v_{t}}{1-\beta_{2}{}^{t}}$$

Finally, we update the parameters as in the following equation, where α is the step size.

(6) $$\theta_{t}=\theta_{t-1}-\alpha \frac{\hat{m_{t}}}{\sqrt{\hat{v_{t}}}+\epsilon }$$

The loss function uses binary cross-entropy and is expressed by the following equation:

(7) $$E=-\underset{k}{\sum }t_{k}\log y_{k}$$

where log is the natural logarithm with base e. Let $y_{k}$ be the output of the neural network, and $t_{k}$ is set to 1 only for the index that is the correct label and 0 otherwise. Therefore, the binary cross-entropy formula given above is for calculating the natural logarithm of the output corresponding to a correct label of 1.

### 2.3. Improved U-Net Architecture

An improved U-Net architecture was obtained by modifying the conventional U-Net architecture shown in Figure 2. A part of this modification was presented in a conference proceeding [13]. In the present paper, we additionally detailed all the sections and added Section 2.4, “Learning with incomplete labeling data”, which was not present in the above-cited conference proceeding.

In the present paper and based on the conference proceeding [13], we propose an improved U-Net architecture by considering what features of the image are learned in what part of the CNN model. Specifically, we visualized the learning process by outputting feature maps and attempted to address the problems associated with the conventional U-Net architecture [14]. As a result, it was found that some areas were not properly segmented for particles with dark interiors, and this was due to the learning result on the encoder side. Therefore, skip connections were removed, and the amount of information on the encoder side was reduced when up-sampling was performed on the decoder side. The differences in features between the U-Net by Olaf et al. [9] and the U-Net proposed in this study are as follows. Compared to the conventional U-Net, the improved U-Net#1 and U-Net #2 have a shorter learning time. Moreover, the improved U-Net #2 removes deep skip connections and reduces weights so that the up-sampling on the decoder side is less affected by the encoder side. This improves the accuracy of region segmentation, especially for particles with extremely dark interiors.

**Figure 3:**
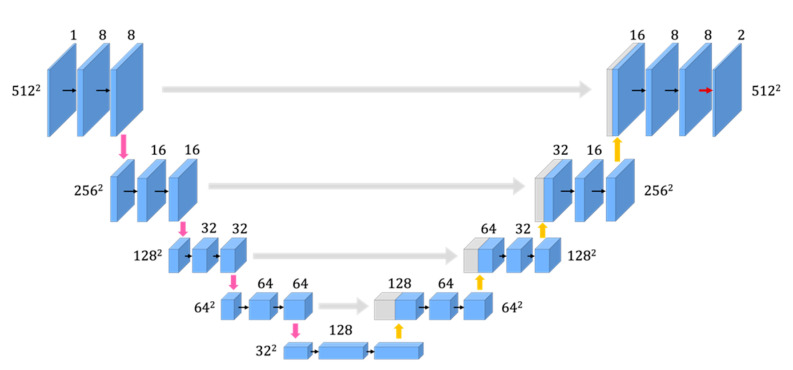
Improved U-Net (U-Net #1) with fewer channels than the conventional architecture.

The size of the image and the number of channels in the conventional U-Net are indicated by the values next to the CNN in Figure 2. The size of the image and the number of channels in the improved U-Net #1 are indicated in Figure 3. The size of the input image in each network architecture is 512 × 512 and is reduced to 32 × 32 using a max pooling layer in the encoder pass. The features can be extracted from the image using these processes. In the decoder pass, the resolution is increased from 32 × 32 to 512 × 512 using an up-sampling layer.

#### 2.3.1. Decreasing the Number of Channels

First, improved U-Net #1 was established with a lower number of channels than the conventional architecture, as shown in Figure 3.

**Figure 4:**
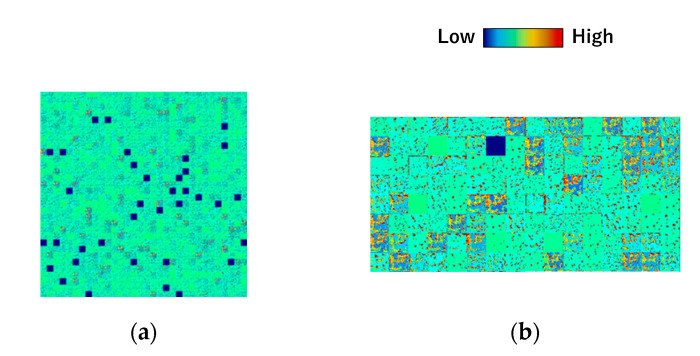
Feature maps of the deep layer (**a**) before (conventional U-Net) and (**b**) after (U-Net #1) the number of channels was decreased.

Figure 4 displays the feature maps considered to establish U-Net #1. Figure 4a shows the deep feature map for learning based on the original U-Net. The weights are not updated by training based on multiple filters. Figure 4b shows the corresponding deep feature map for U-Net #1, which indicates that all the filters are used for learning. These findings indicate that the number of channels in the original architecture was excessively large for the target image. Therefore, the architecture with the decreased number of channels was used in the subsequent analyses.

#### 2.3.2. Improvement of the Deep Layer Architecture

**Figure 5:**
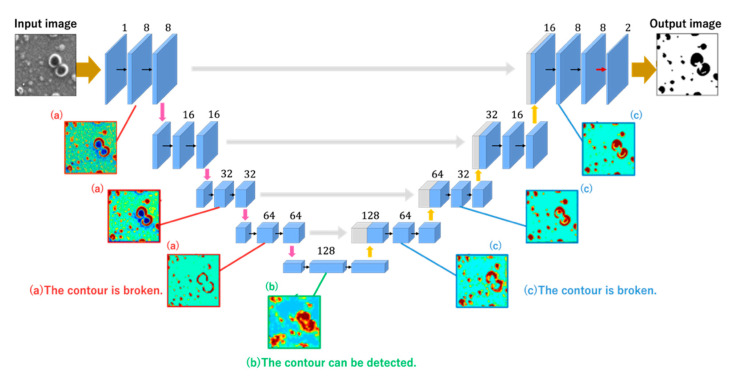
Part of feature maps of the hidden layer in U-Net #1.

Segmentation of particles with extremely dark interiors may not yield correct results. The feature maps shown in Figure 5 were considered to increase the segmentation accuracy of such particles by modifying the U-Net architecture. We found that the decoder side detects broken contours. This is problematic from the viewpoint of accurate segmentation. However, if we observe the feature map outputs at the deepest layers, they demonstrate that contours can be successfully detected, although they are not clearly visible owing to the low resolution. However, the subsequent feature map output by the decoder side shows that the particle contours are again detected as broken in the intermediate layer, and the interior of the particle is not classified as a particle; it appears as if the interior of the particle is missing. Subsequently, the final output of the segmentation results shows that some particle areas have been partially overpainted. We assumed the reason for the final segmentation result to be inaccurate even though the deepest layers successfully detected the particles is that the information in the intermediate encoder layers was merged using skip connections. Therefore, in this study, we reduced the amount of encoder-side information transmitted when combining the encoder-side information through skip connections. This is expected to accurately segment particles with darker interiors and less distinct contours. The skip connections, which are a U-Net feature, directly compensate for the detailed image information lost when the image is scaled down at the encoder side, and this process enables precise domain segmentation. We assumed that the skip connections in the intermediate layer cause the defect in segmentation, and reducing the skip connections in the intermediate layer yield significant segmentation even if high-resolution information is lost.

**Figure 6:**
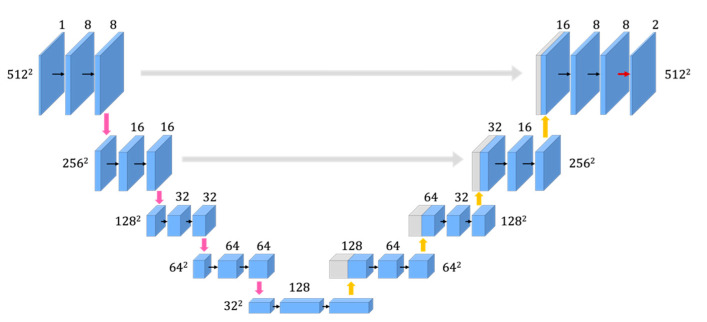
Improved U-Net (U-Net #2): Deleted two skip connections.

In the improved U-Net #2 (Figure 6), we deleted two skip connections that connected the deep layers. These architectures were implemented in Python using Keras [12]. The size of the image and the number of channels in the improved U-Net #2 are indicated in Figure 6.

### 2.4. Learning with Incomplete Labeling Data in Improved U-Net #1

The ground truth mask data used in this study were generated by manually labeling the particle parts based on the microscopic images of the colored material. However, this method for generating the training data is labor intensive. Therefore, the burden of creating labels for the microscopic images of colorants must be decreased.

The microscopic images of colorants include different types of particles, for example, large particles with clear outlines and small particles with ambiguous outlines. The labeling of the former and latter types of particles is less and more labor intensive, respectively. In particular, large particles with clear particles can be instantly identified as particles, whereas the identification of small particles may be time-consuming. In this context, the labeling of small particles is a key reason for the tedious nature of generating the ground truth data. This burden can be alleviated by eliminating the need to label small particles.

Therefore, in this study, we prepared incomplete labeling data without labeling small particles and used these data for training. Subsequently, we attempted to determine the number of small particles to be labeled to achieve accurate segmentation.

**Figure 7:**
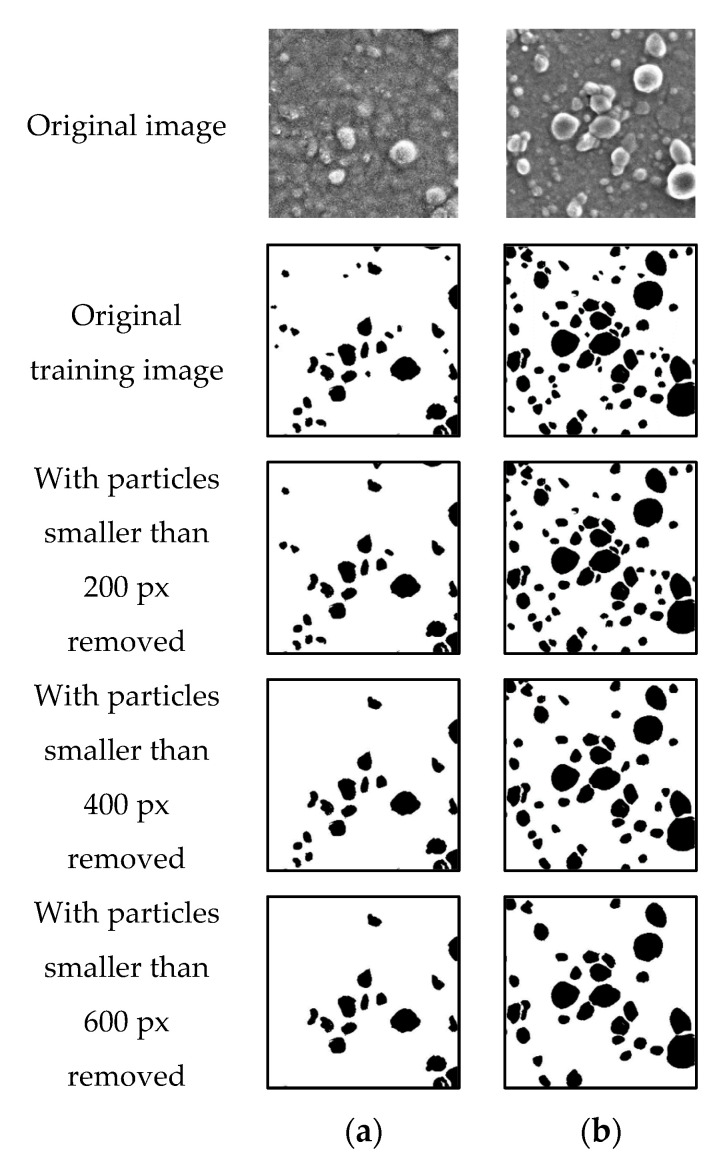
Sample incomplete labeling images with (**a**) small number of particles; (**b**) large number of particles.

The training data were defined as the original training data, which included labels for even the smallest particles, although a certain amount of data was expected to be missing. From these data, we generated the labeling data by deleting particles smaller than 200, 400, 600, 800, and 1000 px in images with a small and large number of particles. MATLAB was used to implement the image processing. The labeling data, as shown in Figure 7, were used to train U-Net #1.

## 3. Results

This section describes the images and accuracy obtained using the method described in Section 2. We used two types of microscopic images of color materials for training: images with small (Figure 1a) and large (Figure 1b) number of particles. Both images were representative of a mixture of carbon black pigment and resin; however, the compositions of the mixtures were different.

The accuracy of the images obtained by training was evaluated. The confusion matrix presented in Table 1 was used to determine the accuracy, recall, and precision, defined as in Equations (8)–(10), respectively [15]. True (TP) and false positive (FP) correspond to cases in which the part classified as a particle and background in the ground truth is classified as a particle in the prediction image, respectively. False negative (FN) and true negative (TN) correspond to cases in which the part classified as a particle and background in the ground truth is classified as the background in the prediction image, respectively [16].

The accuracy is a measure of the percentage of the number of correctly classified data among the total data, that is, the percentage corresponding to TP and TN. The recall indicates the proportion of the number of data that were positive among the data that should be classified as positive, that is, the proportion of TP in the total of TP and FN. The precision is a measure of the percentage of the number of data that are actually positive among the data classified as positive, that is, the percentage corresponding to TP and FP. A higher accuracy, recall, and precision is preferable.

(8) $$Accuracy=\frac{TP+TN}{TP+FP+FN+TN}$$

(9) $$Recall=\frac{TP}{TP+FN}$$

(10) $$Precision=\frac{TP}{TP+FP}$$

Here, accuracy indicates the overall recognition ratio. However, the accuracy alone is insufficient, and additional ratio parameters, such as parameters, precision, recall, and the F1-score evaluation criteria, are required. F1-score is defined as the harmonic mean of the precision rate and the recall rate and is a useful value for balancing the precision and recall metrics [17]. It is calculated as follows:

(11) $$F1=2*\frac{Precision*Recall}{Precision+Recall}$$

### 3.1. Learning Based on U-Net Architectures

The training time for the conventional U-Net was 780 s, but the improved U-Net #1 and improved U-Net #2 present training times of 450 s and 375 s, respectively, on the GPU (NVIDIA GeForce RTX 2080 Ti). The training time was significantly reduced owing to network improvements. The inference based on the learning model can be output in 2–3 s in either network architecture. In the improved U-Net #1, the number of required parameters was reduced by reducing the number of channels without changing the size of the input image, resulting in a dimensionality reduction. Consequently, the learning time is reduced. In the improved U-Net #2, the removal of skip connections in the deeper layers is assumed to have reduced the learning time as fewer processing steps are required.

**Figure 8:**
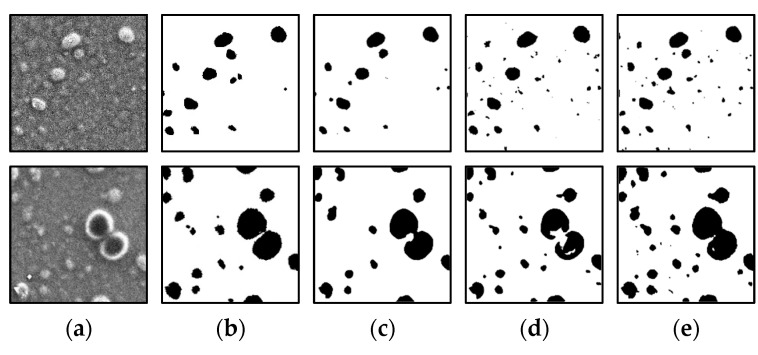
Results of image processing after segmentation (small number of particles). (**a**) Input data; (**b**) Output data (ground truth). Results obtained using (**c**) conventional U-Net; (**d**) U-Net #1; (**e**) U-Net #2.

**Figure 9:**
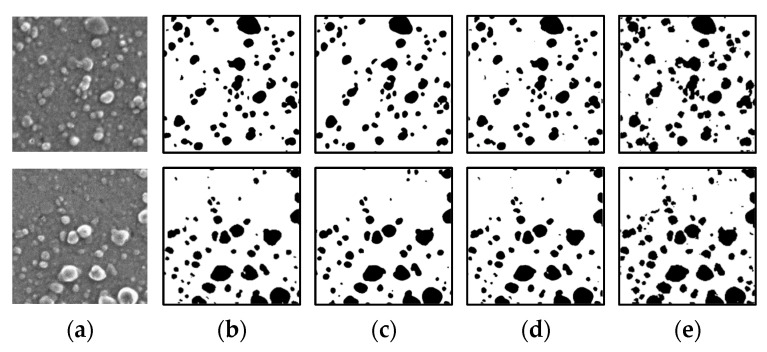
Results of image processing after segmentation (large number of particles). (**a**) Input data; (**b**) Output data (ground truth). Results obtained using (**c**) conventional U-Net; (**d**) U-Net #1; (**e**) U-Net #2.

Figure 8 shows the results of the segmentation of a colorant microscopic image with a small number of particles using the conventional U-Net, U-Net #1, and U-Net #2. In the case of the upper image, the results of all the U-Net architectures are comparable. However, in the case of the lower image, the large particle in the upper right corner is not properly segmented. In particular, the particle part is almost undetectable when the conventional U-Net is used, and the segmentation is slightly enhanced when U-Net #1 and #2 are used. Figure 9 shows the results of the segmentation of a colorant microscopic image with a large number of particles using the conventional U-Net, U-Net #1, and U-Net #2. For the upper and lower images, the results obtained using the conventional U-Net and U-Net #1 are similar. However, several particles are not properly detected by U-Net #2.

Table 2 presents the segmentation accuracies of colorant microscopic images with a small number of particles using different U-Net architectures. The accuracy is comparable for all the U-Net architectures. U-Net #2 has a high recall but small precision compared to the other U-Net architectures. The accuracy of U-Net #1 is similar to that of the conventional U-Net architecture.

Table 3 presents the segmentation accuracies of colorant microscopic images with a large number of particles using different U-Net architectures. The accuracy is comparable for all the U-Net architectures. U-Net #2 has a high recall but small precision compared to the other U-Net architectures. The accuracy of U-Net #1 is similar to that of the conventional U-Net architecture. In particular, the accuracies of U-Net #1 and the conventional U-Net are identical.

In this study, the ground-truth images were manually created and are not perfectly correct data. In many cases, especially for small particles, they are not classified as particles. On the other hand, there are very few areas where non-particles are incorrectly classified as particles. This is because, when creating the ground-truth images, we set a rule that, if it is difficult to determine whether they are particles or not, the parts are not classified as particles. Based on this rule, it is expected that the areas classified as particles at the time of the ground-truth-image creation are almost certainly particles. Therefore, in the accuracy evaluation in this study, priority was given to improving the recall score, which indicates the percentage of particles in the ground-truth images that are properly classified as particles. Comparing the recall scores in Table 2 and Table 3, the values corresponding to U-Net #1 and U-Net #2 are improved over the conventional U-Net.

**Figure 10:**
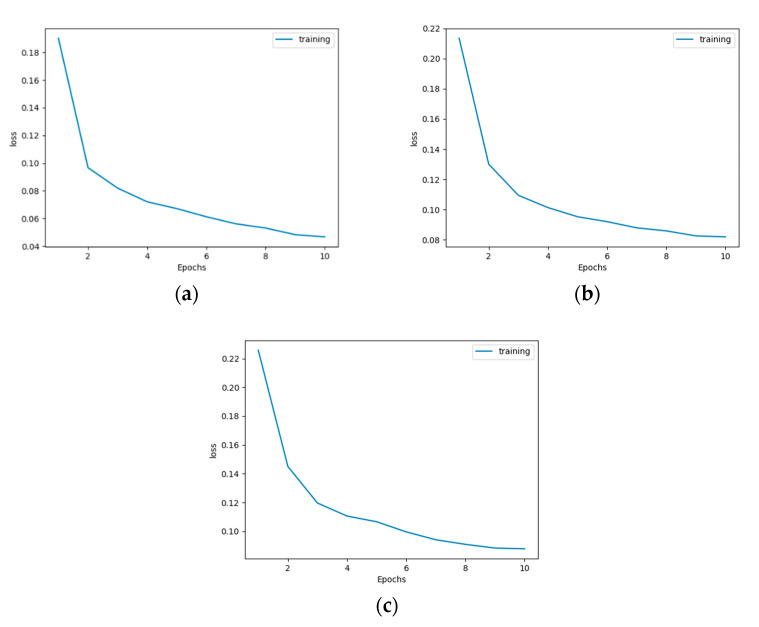
Training curve of (**a**) conventional U-Net, (**b**) improved U-Net #1, and (**c**) improved U-Net #2.

Figure 10a–c illustrates the training curve of each network architecture. Table 2 and Table 3 present the prediction results for the test data. It can be observed that this accuracy is similar to the results of the training data and that our model was not prone to overfitting.

To quantitatively assess the accuracy of the model, comparisons were made using the area under the curve (AUC) of the Receiver Operating Characteristic curve (ROC). Table 4, Table 5 and Table 6 present the confusion matrices corresponding to images with a small number of particles for conventional U-Net, improved U-Net #1, and improved U-Net #2. Table 7, Table 8 and Table 9 present the confusion matrices corresponding to images with a large number of particles for conventional U-Net, improved U-Net #1, and improved U-Net #2. Comparing the values in these tables, an insignificant difference can be observed between the matrices for conventional U-Net, improved U-Net #1, and improved U-Net #2. Therefore, the proposed method does not significantly improve the confusion matrix. However, when comparing Figure 9 and Figure 10, significant results were obtained in terms of the appearance of particle domain segmentation. However, as mentioned above, a comparison of Figure 9 and Figure 10 shows visually that the improved network detects particles more accurately than the conventional U-Net. Therefore, better results were obtained using the improved U-Net in terms of the appearance of the particle area segmentation. Comparing the AUC values shown in Table 10 and Table 11, the accuracy of the improved U-Net #1 is comparable to that of the conventional U-Net. On the other hand, the accuracy of the improved U-Net #2 is slightly lower than that of the conventional U-Net. The results demonstrate that the proposed method maintained the previous high level of accuracy in quantitative evaluation while improving the visual appearance of the particles in the segmentation of the regions of the particles. The number of particles in the image is inversely related to the size of the particles; i.e., the larger the number of particles in the image, the smaller the size of the particles and vice versa. Therefore, the AUC scores shown in Table 10 and Table 11 are close to the human model in that larger particles are easier to distinguish.

We calculated the F1-scores for each network architecture and compared the detection accuracy. Table 12 presents the F1-scores for the conventional U-Net, improved U-Net #1, and improved U-Net #2. The accuracy is better when this score is closer to 1. The comparison of the values in the table shows that the conventional U-Net presents the best score, and the improved U-Net #2 has similar scores. The evaluation based on F1-score indicates that the conventional U-Net and the improved U-Net #2 present similar performance in our application.

### 3.2. Learning U-Net #1 Based on Incomplete Labeling

**Figure 11:**
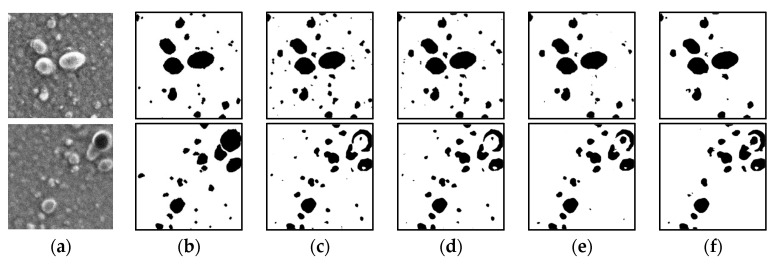
Results of image processing after segmentation (small number of particles). (**a**) Input data; (**b**) Output data (ground truth). Results obtained using (**c**) original ground truth data; and labeling data in which particles smaller than (**d**) 200 px; (**e**) 400 px; and (**f**) 600 px are removed.

Figure 11 displays the segmentation results of a colorant microscope image with a small number of particles with incomplete labeling data used as the ground truth data. The results of training using the original ground truth data and labeled data in which particles smaller than 200 px are removed indicate that despite the presence of noise, the particle regions can be correctly detected. The results of training using labeling data in which particles smaller than 400 px are removed are similar to the correct answers; however, certain small particles are not detected. In the training using labeling data in which particles smaller than 600 px are removed, the number of undetected particles is larger than that in the other cases.

**Figure 12:**
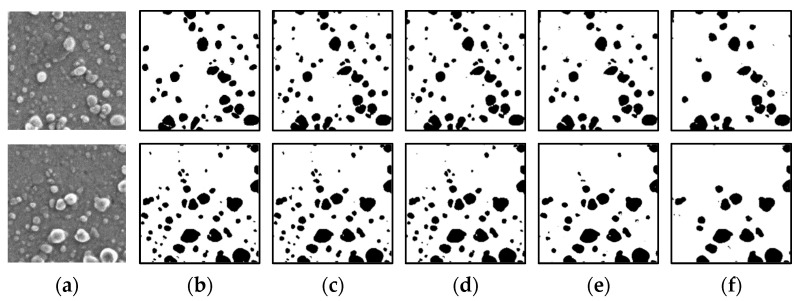
Results of image processing after segmentation (large number of particles). (**a**) Input data; (**b**) Output data (ground truth). Results obtained using (**c**) original ground truth data; and labeling data in which particles smaller than (**d**) 200 px; (**e**) 400 px; and (**f**) 600 px are removed.

Figure 12 shows the segmentation results of a colorant microscope image with a large number of particles, with incomplete labeling data used as the ground truth data. When the training is performed using the original ground truth data and labeled data in which particles smaller than 200 and 400 px are removed, the particle regions are correctly detected. However, several small particles are not detected when labeling data in which particles smaller than 400 px are removed are used. In the training using labeling data in which particles smaller than 600 px are removed, the number of undetected particles is larger than that in the other cases, which is undesirable.

Table 13 and Table 14 present the segmentation-accuracy values obtained using U-Net #1 for colorant microscopic images with a small and large number of particles, respectively, with incomplete labeling data used as the supervised data. The accuracy is comparable for all the labeled data. However, when labeling data in which particles smaller than 400 px and 600 px are removed are used, the precision is higher, and recall is lower.

The F1-score was calculated to compare the accuracy of learning when using incomplete labeling data. As shown in Table 15, the F1-score is highest for learning when using data with particles smaller than 200 px removed, both for a small and large number of particles. This result demonstrates that removing particles smaller than 200 px when creating the ground-truth images does not affect the learning results and simplifies the creation of the dataset.

## 4. Discussion

We segmented two types of colorant microscopic images using improved U-Net architectures. Subsequently, we evaluated the accuracy of the segmented images obtained by the training. This study focused on avoiding any missed detection of particles. Based on this idea and the resulting training images, the improved U-Net achieved significant accuracy for both color material microscope images with a small number of particles and color material microscope images with a large number of particles. For particles with darker interiors, the accuracy of region segmentation was improved by removing skip connections. This study focused on avoiding any missed detection of particles.

To verify the simplicity of labeling in the creation of the ground truth data, we used the incomplete labeling data as the ground truth data and segmented the two types of colorant microscopic images. The resulting images indicate that the segmentation accuracy is significantly reduced when the labeling data in which particles smaller than 600 px are removed are used. Moreover, the recall decreases when the labeling data in which particles smaller than 400 px and 600 px are removed are used. Because this study focused on avoiding misdetection, the low recall is undesirable. The results obtained using the labeling data in which particles smaller than 200 px are removed are equivalent to those obtained using the original ground truth data. This finding indicates that, in learning with U-Net #1, the segmentation results are not adversely influenced even when particles smaller than 200 px are not labeled during the creation of the ground truth data.

## 5. Conclusions

We used improved U-Net architectures to segment two types of colorant microscopic images. We verified the accuracy of the results obtained using the U-Net architectures using the following metrics: accuracy, recall, and precision. U-Net #1 exhibited the highest accuracy for colorant microscopic images with a small and large number of particles, respectively. To verify the simplicity of the labeling process when creating ground truth data for training, we used incomplete labeling data for training. For both types of colorant microscopic images, the results obtained using labeling data with particles smaller than 200 px removed and original ground truth data were comparable.

Future work can focus on two aspects: first, it is necessary to perform training over a wider range of colorant microscopic images to achieve a high accuracy segmentation to enhance the versatility of the proposed method. Second, it is necessary to separate and segment agglomerated particles. We observed that several agglomerated particles were segmented as a single coherent particle in this study, which is undesirable for measuring the particle size distribution. By separating the agglomerated particles and segmenting them, accurate particle size distribution can be obtained, which is expected to be of practical use. Third, because this research is a preliminary study to observe if changing the structure is practical in our application, we will extend this research to formulate a general method for practical use in region segmentation of color microscope images.

## Figures and Tables

**Table 1:** Confusion matrix.

		Model Prediction	
		Positive	Negative
**Class**	**Positive**	TP (True Positive)	FN (False Negative)
	**Negative**	FP (False Positive)	TN (True Negative)

**Table 2:** Accuracy, recall, and precision (image with small number of particles).

	Accuracy	Recall	Precision
Conventional U-Net	0.944	0.598	0.691
U-Net #1	0.940	0.626	0.632
U-Net #2	0.921	0.717	0.486

**Table 3:** Accuracy, recall, and precision (image with large number of particles).

	Accuracy	Recall	Precision
Conventional U-Net	0.879	0.685	0.682
U-Net #1	0.879	0.686	0.681
U-Net #2	0.854	0.766	0.569

**Table 4:** Confusion matrix (Conventional U-Net/ small number of particles).

		Model Prediction	
		Positive	Negative
**Class**	**Positive**	0.04976590	0.03345044
	**Negative**	0.02226791	0.89451573

**Table 5:** Confusion matrix (Improved U-Net #1/ small number of particles).

		Model Prediction	
		Positive	Negative
**Class**	**Positive**	0.05446129	0.02152138
	**Negative**	0.05766894	0.86634839

**Table 6:** Confusion matrix (Improved U-Net #2/ small number of particles).

		Model Prediction	
		Positive	Negative
**Class**	**Positive**	0.05143394	0.03006414
	**Negative**	0.03215675	0.88634516

**Table 7:** Confusion matrix (Conventional U-Net/ large number of particles).

		Model Prediction	
		Positive	Negative
**Class**	**Positive**	0.13076969	0.06001841
	**Negative**	0.06108787	0.74812419

**Table 8:** Confusion matrix (Improved U-Net #1/ large number of particles).

		Model Prediction	
		Positive	Negative
**Class**	**Positive**	0.13754247	0.04195480
	**Negative**	0.10402209	0.71648064

**Table 9:** Confusion matrix (Improved U-Net #2/ large number of particles).

		Model Prediction	
		Positive	Negative
**Class**	**Positive**	0.13199094	0.05763283
	**Negative**	0.06635412	0.74402212

**Table 10:** AUC (small number of particles).

Conventional U-Net	Improved U-Net #1	Improved U-Net #2
0.92612012	0.91877028	0.89801622

**Table 11:** AUC (large number of particles).

Conventional U-Net	Improved U-Net #1	Improved U-Net #2
0.90651809	0.90661659	0.89874124

**Table 12:** F1-score for each network architecture.

	Small Number of Particles	Large Number of Particles
Conventional U-Net	0.57481953	0.63817387
U-Net #1	0.46757251	0.57124212
U-Net #2	0.51764668	0.60841551

**Table 13:** Accuracy, recall, and precision (small number of particles).

	Accuracy	Recall	Precision
Original	0.940	0.626	0.632
Particles smaller than 200 px removed	0.945	0.607	0.668
Particles smaller than 400 px removed	0.945	0.547	0.733
Particles smaller than 600 px removed	0.942	0.501	0.736

**Table 14:** Accuracy, recall, and precision (large number of particles).

	Accuracy	Recall	Precision
Original	0.879	0.686	0.681
Particles smaller than 200 px removed	0.879	0.690	0.680
Particles smaller than 400 px removed	0.882	0.645	0.718
Particles smaller than 600 px removed	0.874	0.569	0.739

**Table 15:** F1-score for learning with incomplete labeling data.

	Small Number of Particles	Large Number of Particles
Original	0.62898569	0.68349086
Particles smaller than 200 px removed	**0.63604078**	**0.6849635**
Particles smaller than 400 px removed	0.62648594	0.67954512
Particles smaller than 600 px removed	0.59617785	0.6429526
